# EBE-YOLOv4: A lightweight detecting model for pine cones in forest

**DOI:** 10.3389/fpls.2022.1042332

**Published:** 2022-11-11

**Authors:** Zebing Zhang, Dapeng Jiang, Huiling Yu, Yizhuo Zhang

**Affiliations:** ^1^ School of Computer Science and Artificial Intelligence, Changzhou University, Changzhou, Harbin, China; ^2^ School of Mechanical and Electrical Engineering, Northeast Forestry University, Changzhou, Harbin, China

**Keywords:** pine cones detection, YOLOv4, EfficientNet-b0, BiFPN, ECA-Net, Hard-Swish

## Abstract

Pine cones are important forest products, and the picking process is complex. Aiming at the multi-objective and dispersed characteristics of pine cones in the forest, a machine vision detection model (EBE-YOLOV4) is designed to solve the problems of many parameters and poor computing ability of the general YOLOv4, so as to realize rapid and accurate recognition of pine cones in the forest. Taking YOLOv4 as the basic framework, this method can realize a lightweight and accurate recognition model for pine cones in forest through optimized design of the backbone and the neck networks. EfficientNet-b0 (E) is chosen as the backbone network for feature extraction to reduce parameters and improve the running speed of the model. Channel transformation BiFPN structure (B), which improves the detection rate and ensures the detection accuracy of the model, is introduced to the neck network for feature fusion. The neck network also adds a lightweight channel attention ECA-Net (E) to solve the problem of accuracy decline caused by lightweight improvement. Meanwhile, the H-Swish activation function is used to optimize the model performance to further improve the model accuracy at a small computational cost. 768 images of pine cones in forest were used as experimental data, and 1536 images were obtained after data expansion, which were divided into training set and test set at the ratio of 8:2. The CPU used in the experiment was Inter Core i9-10885@2.40Ghz, and the GPU was NVIDIA Quadro RTX 5000. The performance of YOLOv4 lightweight design was observed based on the indicators of precision (P), recall (R) and detection frames per second (FPS). The results showed that the measurement accuracy (P) of the EBE-YOLOv4 was 96.25%, the recall rate (F) was 82.72% and the detection speed (FPS) was 64.09F/S. Compared with the original YOLOv4, the precision of detection had no significant change, but the speed increased by 70%, which demonstrated the effectiveness of YOLOv4 lightweight design.

## 1 Introduction

As an important forest product, Korean pine cone has high edible and medicinal value. At present, pine cone picking is mainly realized by people climbing trees to knock them down, and then collecting them on the ground. Because pine cones are small and scattered in the forest, and their color is close to the ground after landing, the whole picking process is time-consuming and labor-intensive. In the process of pine cone picking, there are not only potential dangers but also the possibility of missing the pine cones. Therefore, a pine cone identification method based on machine vision is necessary. On the one hand, it can be integrated into the drone to realize the observation of pine cones and guide manual picking in forest and monitor the growth state of pine cones and guide the cultivation of Korean pine; on the other hand, the method can be integrated into ground devices for automatic collection of fallen pine cones. Therefore, it has important application value to study a fast and accurate identification method of pine cones in forest.

Pine cone detection falls into the category of small target detection, a topic has been receiving extensive attention from scholars in recent years. Related researchers have developed from traditional methods based on shape, color and texture features to convolutional networks (Fang et al., 2018). For grape picking, Rodrigo et al selected HOG (Histogram of Oriented Gradients) and LBP (Local Binary Pattern) to extract shape and texture features of grapes, and then used SVM-RBF to build a grape recognition classifier (Perez-Zavala et al., 2018). For apple harvesting, Zartash et al used HS model to locate and segment the apple images, and then used refinement denoising and Hough transform to realize accurate location of the apples (Kanwal et al., 2019). Gu Suhang et al introduced the ASIFT feature to repair the target hollow areas generated by K-means clustering, and used the gPb contour detector and the dynamic threshold Otsu method successively to generate clear and continuous target contours (Gu et al., 2017). In order to improve the detection accuracy of traditional detection methods, the design requires an organic combination of multiple methods, which requires cumbersome steps and complex procedures. As target detection based on deep learning gradually becomes a research hotspot, Lin et al used Faster RCNN to detect strawberry flowers in various environments, with an accuracy rate of 86.1 (Lin et al., 2020). He et al proposed Mask-RCNN on the basis of Faster RCNN, adding a prediction branch and replacing ROI pooling with ROI Align, which improved the detection accuracy (He et al., 2020). Yue Youjun et al used Mask-RCNN optimized by the boundary weighted loss function to realize the recognition and localization of apples in complex environments (Yue et al., 2019).

The two-stage algorithm has high detection accuracy, good versatility and robustness, but the training and detection process takes a long time. For this, Redmon et al proposed YOLOv1 (Redmon et al., 2016), which discards the candidate frame generation structure and combines feature extraction, candidate frame classification and regression in an end-to-end network. YOLOv1 improved the detection rate significantly compared with the two-stage algorithm, but its detection accuracy was reduced. After that, Redmon proposed YOLOv2 (Redmon and Farhadi, 2017), using a new feature extraction network DarkNet19, introducing a batch normalization (BN) layer to enhance the network convergence speed, and using k-means clustering algorithm to automatically find prior anchor boxes, thereby improving detection performance. Redmon also proposed YOLOv3 (Redmon and Farhadi, 2018), which designed a DarkNet53 full convolution network without fully connected layer, combined with the FPN idea, and fused feature maps of three scales to improve target detection performance. On the basis of YOLOv3, Bochkovskiy et al proposed the YOLOv4 detection model by combining data enhancement Mosaic method, CSP feature extraction module, spatial feature pyramid (SPP), PANet feature fusion and other improved ideas, and the detection accuracy was further improved (Bochkovskiy et al., 2020). However, the CSP-DarkNet53 backbone network and the PANet feature fusion structure require a large amount of computation, which lead to the reduction of model computational efficiency.

General detection models often require a large number of parameters and thus result in high computational complexity, therefore, they cannot meet the requirement of real-time required by embedded devices and thus requires lightweight improvement. Wang et al proposed YOLOv4-tiny, which reduces the number of detection branches by compressing the network depth, and uses strategies such as FPN feature fusion structure to reduce the amount of computation and improve the operation efficiency (Wang et al., 2021). Fu Huitong et al used a lightweight GhostNet network module to reconstruct the backbone network for the large computational load of YOLOv4 (Fu et al., 2021). Ye Zixun et al proposed a lightweight MN3-YOLOv4-Lite model (Ye and Zhang, 2021), which took MobileNetv3 as the backbone network and replaced the ordinary convolution in the 3-layer and 5-layer convolution blocks with the depthwise separable convolution, realizing the lightweight network. EfficientNet (Tan and Le, 2019) combines the advantages of various feature extraction networks, and then used grid search method to determine the optimal structure, and achieved better detection effect with less computational cost. Efficientnet series has many versions. Among them, b0 is the basic one, which performs well in both accuracy and efficiency. Other versions of Efficient stack the convolution layers to improve the accuracy. Although the accuracy is slightly improved, the amount of calculation is increased by geometric multiples, which is not conducive to real-time detection. Subsequently, EfficientDet (Tan et al., 2020) introduced an efficient BIFPN feature fusion structure, through cross-layer connection and changing concating into adding, which reduced the amount of computation. In order to improve the accuracy of the model, Li Mukai et al (Li et al., 2020) introduced the SENet (Hu et al., 2020) attention mechanism in YOLOv3 to detect small pedestrian targets in infrared images. The accuracy and recall rate were both improved, but the two fully connected layers were computationally intensive. SGE-Net (Li et al., 2019)expressed different semantic features by grouping sub-features on the basis of SE-Net, so as to learn and suppress noise in a more targeted manner. Efficient Channel Attention Network (ECA-Net) (Wang et al., 2020) reduced the amount of computation significantly by introducing a more efficient 1 D-convolution for local feature interaction.

To sum up, among many target detection algorithms, the two-stage algorithm, represented by fast RCNN, has high detection accuracy, but its efficiency is low. The efficiency of the YOLO algorithm is better than the two-stage algorithm as a whole, but the detection accuracy is not as good as the former. With the improvement of the YOLO version, the detection accuracy is gradually improved, and the complexity of the algorithm is also increased. Yolov4 takes into account both detection accuracy and efficiency, but the efficiency still needs to be improved. Aiming at real-time detection of small pinecone targets, this paper uses YOLOv4 as the framework of the detection model, and implements the lightweight design to solve the problems of low computational efficiency and poor real-time performance. In terms of lightweight design, the input end uses EfficientNet-b0 as the feature extraction network, and the neck adopts the channel-transformed BiFPN structure for feature fusion; in terms of improving network accuracy, the neck is embedded in the ECA-Net attention module after feature fusion, and the H-Swish activation function is used to optimize the performance of the model. The main contribution are as follows:

- The lightweight Efficientnet-b0 and the BIFPN feature fusion structure with channel change are introduced for lightweight improvement, which reduces model parameters and improves detection efficiency.
- Aiming at the problem that the lightweight of the model leads to the decline of detection accuracy, the lightweight attention mechanism ECA net and the efficient activation function H-Swish are introduced, which improves the detection accuracy to the greatest extent by introducing a few parameters.
- A novel lightweight network EBE-YOLOv4 is constructed, which combines Efficientnet-b0, BIFPN, and ECA net, and applied to the pine cone detection in the forest. Through collecting and expanding the sample set, the test results after network training proved the effectiveness of the lightweight model.

The other parts of the paper are as follows: Section 2 is the improved method of the lightweight detection model for pine cones in forest, Section 3 is the experimental results and discussion, and Section 4 is the conclusion and prospect.

## 2 Pine cones detection model

### 2.1 The structure of YOLOv4

**Figure 1:**
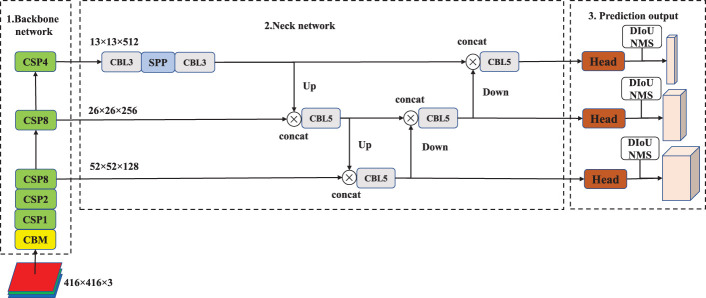
The structure of YOLOv4.

YOLOv4 is divided into 3 sections, and its structure is shown in **Figure 1**. They are the backbone network for feature extraction, neck network for feature fusion, and prediction output. The backbone network is CSPDarkNet53, which consists of 1 CBM and 5 CSP modules. In the backbone network, Mish activation function is introduced into the basic convolution structure, as shown in “CBM” in **Figure 1**. The CSP feature fusion structure, which is composed of three convolutional layers and some Res residual modules, is adopted to enhance the feature extraction ability. Among the 5 CSP modules, the number of Res residual modules is 1, 2, 8, 8, and 4, respectively. P3, P4 and P5 are the three feature layers of YOLOv4. P3 is obtained after three down samples of the original image, P4 is obtained after four down samples, and P5 is obtained after five down samples. The SPP module is introduced in the neck network to promote the information extraction of small-size features, and the PANet of bidirectional feature fusion is introduced to enhance the extraction of underlying feature information. Among them, CBL3 and CBL5 are stacks of 1×1 and 3×3 convolutional layers, which are stacked 3 and 5 times, respectively. In addition, the CIoU Loss function and the DIoU NMS algorithm (Zheng et al., 2020) are used in model training and output prediction.

### 2.2 Lightweight structure design based on YOLOv4(EBE-YOLOv4)

**Figure 2:**
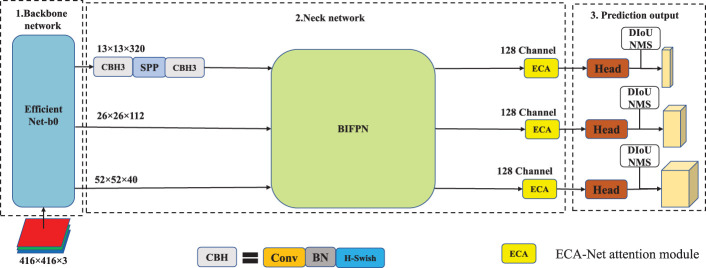
The network structure of EBE-YOLOv4.

As is shown in **Figure 2**, in Lightweight improvement strategy, the backbone network adopts EfficientNet-b0 to achieve a balance between feature extraction capability and computational efficiency. The feature fusion module of neck network adopts the BiFPN structure and undergoes channel transformation to facilitate feature fusion. In addition, in order to improve the detection accuracy after lightweight improvement, the lightweight channel attention module, ECA-Net, is embedded to improve the detection accuracy. Finally, the neck network adopts the H-Swish activation function to fine-tune the model for better detection performance.

#### 2.2.1 EfficientNet-b0

**Figure 3:**
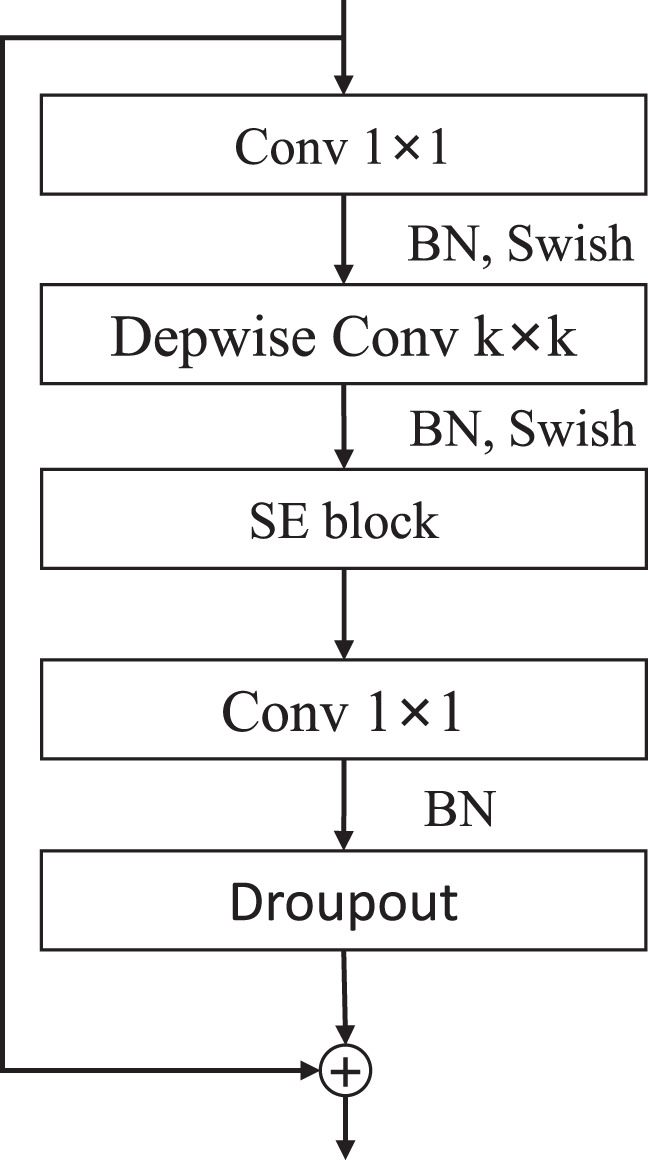
MBConv module structure.

EfficientNet (Wang et al., 2020) uses the Auto ML method to optimize the network structure, strike a balance in network depth, network width and image resolution, reduce model parameters, and improve detection efficiency. The basic structure of the network adopts the mobile inverted residual convolution (MBConv) to achieve better detection results with the least amount of parameters. As shown in **Figure 3**, the first 1×1 Conv realizes the function of increasing the dimension, and the second 1×1 Conv completes the function of reducing the dimension. Six MBConv modules are stacked to form the feature extraction structure MBConv6, the parameter design of each stage is shown in **Table 1**.

**Table 1:** The structure of EfficientNet-b0 network.

Stages	Operator	Output size	Output channel	Repeat
1	Conv3×3	208×208	32	1
2	MBConv1 k3×3	208×208	16	1
3	MBConv6 k3×3	104×104	24	2
4(out)	MBConv6 k5×5	52×52	40	2
5	MBConv6 k5×5	26×26	80	3
6(out)	MBConv6 k5×5	26×26	112	3
7	MBConv6 k5×5	13×13	192	4
8(out)	MBConv6 k3×3	13×13	320	1

The “out” represent feature output channels.

#### 2.2.2 BiFPN feature fusion

**Figure 4:**
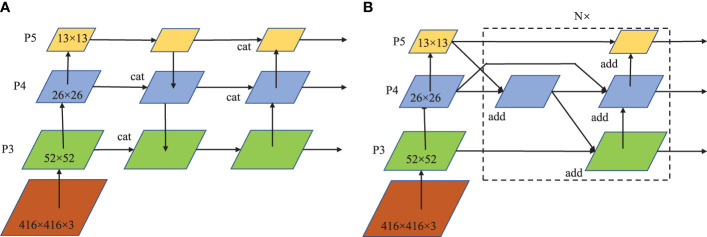
Schematic diagram of feature fusion: **(A)** PANet feature fusion; **(B)** BiFPN feature fusion.

The feature fusion method of YOLOv4 adopts the PANet structure, as shown in **Figure 4A**. The whole structure is divided into two feature aggregation paths from top to bottom and bottom to top respectively, and uses the concatenation method to fuse features which causes the sharp increase in the number of feature channels and the large amount of calculation. Here, the BiFPN feature fusion structure is introduced to reduce the amount of computation. On the basis of PANet, BiFPN deletes the intermediate nodes between the top input and the bottom input, and introduces cross-layer connections to simplify the network structure. In addition, the feature fusion of the weighted addition is adopted, which not only solves the problem of large amount of computation caused by the surge in the number of channels, but also increases the weight of important channels to improve the detection accuracy. The original BiFPN is a 5-layer feature structure. In this study, in order to simplify the parameters and match the YOLOv4 structure, the number of feature layers is reduced to 3, as shown in **Figure 4B**.

The front end of the BiFPN is the channel transfer module to reduce the computation. As shown in **Table 2**, The four transfer channel combinations on P3,P4,and P5 are designed for the BiFPN structure. Group 1 is the original YOLOv4 channel number; the number of channels between different groups is halved in turn from Group 2 to Group 4. Through experimental comparison, the structure of channel 3 is optimized to be the final BiFPN feature fusion channel.

**Table 2:** The transfer channel of BiFPN.

Transfer channel	P3	P4	P5
1	128	256	512
2	256	256	256
3	128	128	128
4	64	64	64

#### 2.2.3 ECA-Net attention

**Figure 5:**
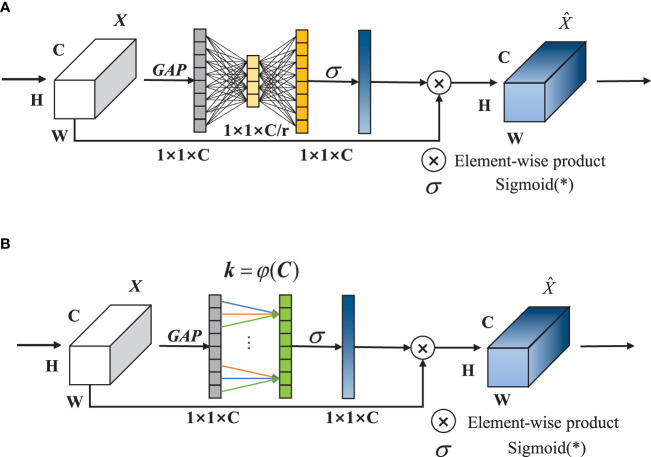
The structures of SE and ECA: **(A)** SE-Net module; **(B)** ECA-Net module.

Ordinary SE-Net channel attention is divided into two sections: squeezing and excitation. In the squeezing section, the input features of a 1D-vector are obtained through global average pooling (GAP). As is shown in **Figure 5A**, In the excitation section, the two-layer fully connected layer (FC) reduces the dimension first and then increases the dimension and obtains the weight of each channel through Sigmoid, which is multiplied with the original feature map and then output. The dimensionality reduction operation is not conducive to the capture of channel features, and the fully connected layer includes a large amount of parameters, which affects the real-time performance. To solve the problems above, ECA-Net is adopted to discard the dimensionality reduction and dimensionality increase operations, and use 1D-convolution instead of double fully connected layers to perform local feature interaction and reduce the amount of calculation. The structure is shown in **Figure 5B**.

Among them, *k*, the size of 1D convolution kernel, is related to the number of channels *C*, as shown in Equation(1).


(1) $$k=\varphi \left(C\right)={\left|\frac{{\text{log}}_{2}\left(C\right)+1}{2}\right|}_{ood}$$

Where, ood means to take the nearest odd number. In order to reduce the padding value in the convolution, the smaller odd number is generally taken.

#### 2.2.4 H-Swish activation function

Swish activation function is shown in Equation(2). Due to the high computational complexity of the Sigmoid function, the gradient is prone to disappear during backpropagation, which leads to information loss. Hard-Swish (H-Swish) introduces Relu6, which reduces the computational cost and avoids gradient vanishing and gradient explosion, defined as Equation(3).


(2) Swish=x∗Sigmoid(βx)

(3) $$H-Swish=x*\frac{Relu6(x+3)}{6}$$

**Figure 6:**
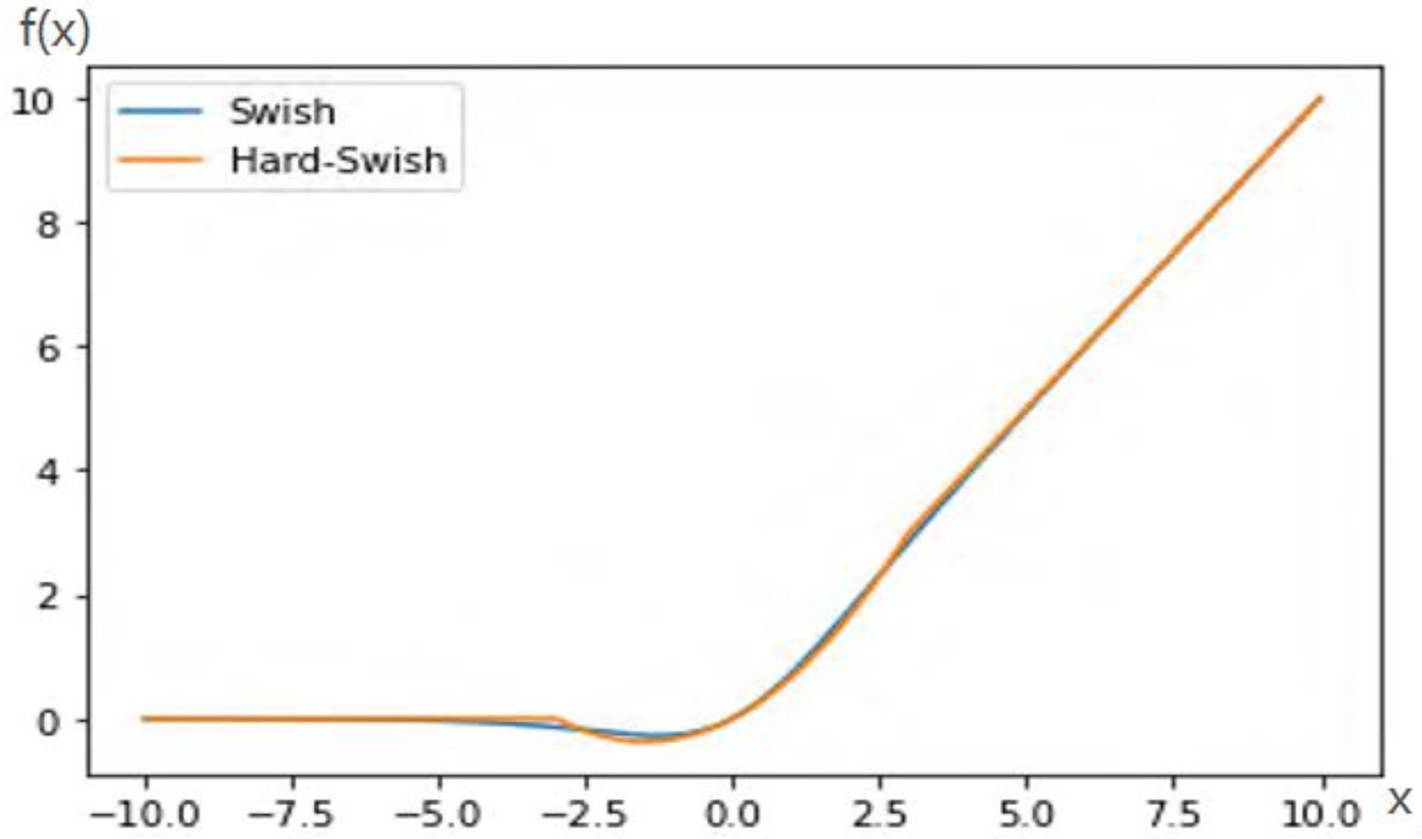
Swish and H-Swish.

**Figure 6** shows the curves of H-Swish and Swish. It is seen from the figure that their trajectories are approximately coincident. Therefore, H-Swish can replace the Swish function.

#### 2.2.5 Loss function

The loss function of YOLOv4 in network training is composed of three parts: confidence loss, boundary loss and classified loss. If there is no objective in a certain boundary, only confidence loss is calculated, and if there is an objective, three kinds of losses are calculated. Loss function expression is shown in formula (4).


(4) $$\left\{ \begin{array}{l} LOSS=L_{ciou}+L_{conf}+L_{class}\\ L_{ciou}=\sum_{i=0}^{s^{2}}\sum_{j=0}^{B}I_{i,j}^{obj}[1-IoU+\frac{\rho^{2}(b,b^{gt})}{c^{2}}+av],(a=\frac{4}{\pi^{2}}{\left(\arctan\frac{w^{gt}}{h^{gt}}-\arctan\frac{w}{h}\right)}^{2},v=\frac{v}{(1-IoU)+v})\\ L_{conf}=-\sum_{i=0}^{S^{2}}\sum_{j=0}^{B}I_{i,j}^{obj}[\hat{C_{i}^{j}}\log(C_{i}^{j})+(1-\hat{C_{i}^{j}})\log(1-C_{i}^{j})]-\lambda_{noobj}\sum_{i=0}^{S^{2}}\sum_{j=0}^{B}I_{i,j}^{noobj}\hat{C_{i}^{j}}\log(C_{i}^{j})+(1-\hat{C_{i}^{j}})\log(1-C_{i}^{j})\\ L_{class}=-\sum_{i=0}^{S^{2}}I_{i,j}^{obj}\sum_{c\in classes}\left[\hat{P_{i}^{j}}\log(P_{i}^{j})+(1-\hat{P_{i}^{j}})\log(1-P_{i}^{j})\right] \end{array}\right.$$

In the formula, *S* ^2^ and *B*:Feature map scale and prior frame; *λ*_*noobj*_ Weight factor; $I_{i,j}^{obj},I_{i,j}^{noobj},i,j$ :If there is a target at the first box of the grid, take 1 and 0 respectively, and if there is no target, take 0 and 1 respectively; *ρ*():Euclidean distance; *c*:The diagonal distance between the predicted box and the actual box closure area; *b*,*w*,*h* :central coordinates and width of the prediction box; b^*gt*^,*w* ^*gt*^,*h* ^*gt*^ :center coordinates and width height of the actual frame; $C_{i}^{j},{\hat{C}}_{i}^{j}$ :confidence levels of the prediction and tagging boxes; $P_{i}^{j},{\hat{P}}_{i}^{j}$ Category probability of prediction box and annotation box.

The confidence loss and classification loss are calculated by cross-entropy method, and the boundary box regression loss is calculated by CIoU loss function. Compared with the traditional mean square error loss function, the problem of sensitivity to the scale of the target object is CIoU effectively avoided.

## 3 Experiment and result analysis

### 3.1 Data set and data amplification

This paper takes the Korean pine cones as the research object, and carries out the detection research of the pine cone in the forest environment. Because the pinecones we studied are almost mature, the color and size of the pinecones are basically similar. A dataset of pine cone images was established through camera field shooting, and the collection location was Jiamusi Forest Farm, Heilongjiang, China. Images were acquired in July, 2019. Data collection includes two scenarios: landing and tree. In the ground pine cone scene, the background color changes obviously. We pay attention to the close shot, long shot, dark environment, etc. Shooting from different angles in 3 time periods during the day, the shooting distance is 3-10m, and a total of 768 images were shot with a resolution of 5312×2988 and labeled with LabelImg. The size of the pine cone fruit in the dataset is between 25×25 and 600×600 pixels, mainly about 300×300, accounting for about 0.57% of the image area, which is a small target that is difficult to detect.

**Figure 7:**
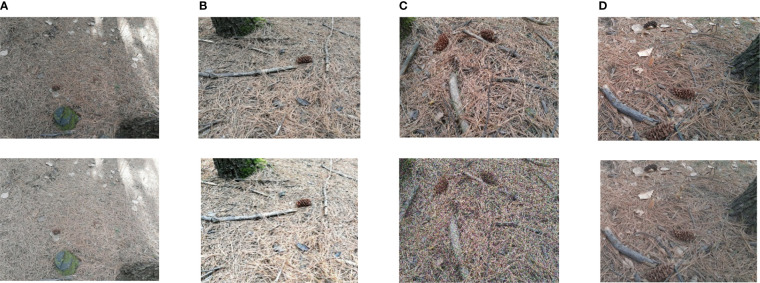
Part of the original image and the image after data processing: **(A)** Contrast Transform; **(B)** random color; **(C)**Gaussian noise; **(D)** salt and pepper noise.

In order to improve the richness of image data and enhance the generalization ability of the model, the dataset was amplified. Contrast transformation, random color, Gaussian noise and salt-and-pepper noise were performed to increase 768 images, totaling 1536 images of pine cones in forest. **Figure 7** shows the pictures of the pine cone and the corresponding pictures after amplification. The images in the first line are the original ones, and the images in the second line are those obtained through the corresponding processing.

### 3.2 Model training and evaluation

The experimental environment is Windows10 system, Pytorch deep learning framework. Hardware equipment CPU is Inter Core i9-10885 @2.40Ghz, memory 64GB, graphics card is NVIDIA Quadro RTX 5000, video memory 16GB. 1536 images are divided into training set and test set according to the ratio of 8:2.

In the network training stage, mosaic data enhancement operation is started to improve the generalization of the model. In order to reduce the cost of the training time, part of the network layer is frozen first, and then the whole network is unfrozen for training. Considering that the training process adopts the “freeze and thaw” strategy, the cosine annealing learning rate does not start, but the learning rate random decay strategy is adopted. The image input size is 416×416×3. Due to the Adam optimizer, the convergence speed is very fast, and the training time is set very short, which is 150 epochs. The first 60 epochs are frozen training, and the initial learning rate is 0.001. the last 90 epochs are thawing training, the initial learning rate is reduced to 5×10^-5^. The larger the Batch Size, the better the model generalization, but it has higher requirements on GPU computing resources. Finally, the Batch Size is set to 8.

Precision rate (P), recall rate (R), AP, F1 and Matthews correlation coefficient (Mcc) are used to evaluate the model detection accuracy, Where AP is the area of P-R curve, F1 reflects the balance of P and R. The calculation is shown in Equations (5-9); the model detection speed is measured by the inference time(IT) and its reciprocal, the number of detected pictures per minute(FPS).


(5) $$P=\frac{N_{TP}}{N_{TP}+N_{FP}}$$

(6) $$R=\frac{N_{TP}}{N_{TP}+N_{FN}}$$

(7) $$AP=\int_{0}^{1}P(R)dR$$

(8) $$F1=\frac{2*P*R}{P+R}$$

(9) $$Mcc=\frac{N_{TP}*N_{TN}-N_{FP}*N_{FN}}{\sqrt{\left(N_{TP}+N_{FP})(N_{TP}+N_{FN})(N_{TN}+N_{FP})(N_{TN}+N_{FN}\right)}}$$

Where, *N_TP_* is the number of positive samples that are detected as positive samples, *N_FP_* is the number of negative samples that are incorrectly classified as positive samples, and *N_FN_* is the number of positive samples that are not detected as positive samples (positive samples are the pine cone areas in the picture, and negative samples are background regions).

### 3.3 Results and analysis

For the above lightweight improvements, the corresponding experiments are designed. The experiments are divided into three parts. First, the effectiveness of EBE-YOLOv4 in pine cones detection is analyzed. Then, the ablation experiments of lightweight optimization process are designed, and the speed and accuracy are tested respectively. Finally, the advantages of EBE-YOLOv4 are verified by comparing with other lightweight networks

#### 3.3.1 The test of EBE-YOLOv4

**Figure 8:**
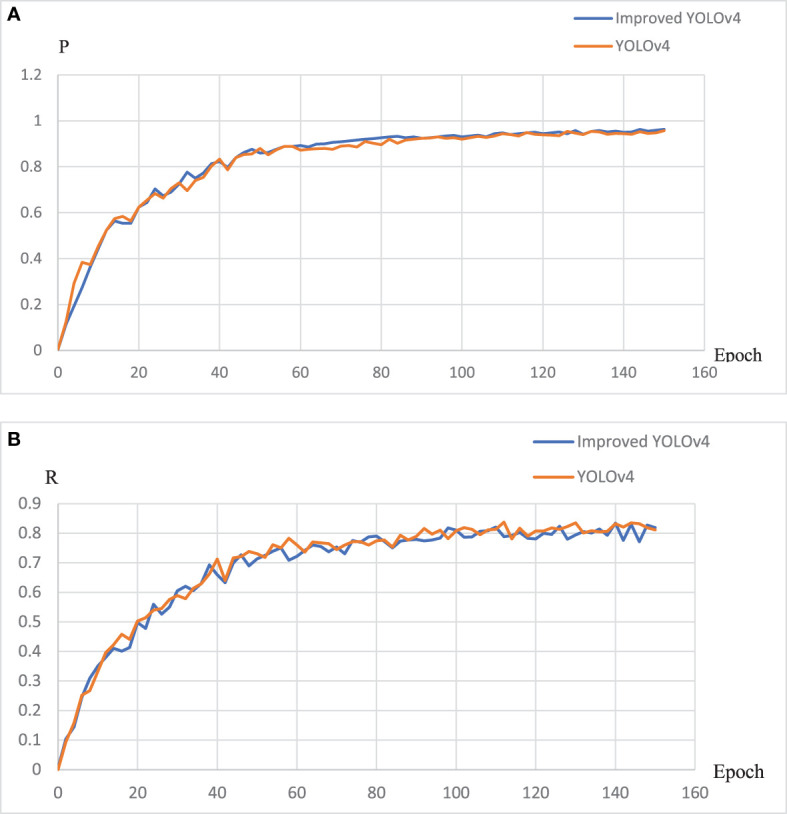
The changes of the P and R indicators during the training process:**(A)** P curve;**(B)** R curve.

**Figure 9:**
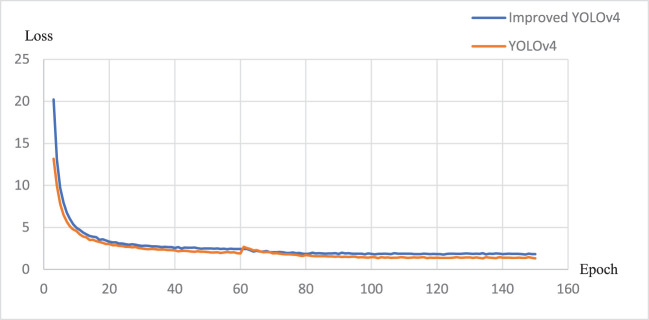
Loss changes during training.

**Figures 8**, **9** present the parameter change curves of YOLOv4 and EBE-YOLOv4 during the training process respectively. It is seen from the figures that the precision rate (P) of the two models is basically the same in the later stage of training, and the recall rate (R) of YOLOv4 is slightly higher than that of the improved lightweight model, but the difference is not obvious. In addition, the lightweight process also affects the loss of the model, making the Loss of the EBE-YOLOv4 model slightly higher than that of YOLOv4, which may directly affect the confidence of pinecone detection.

**Figure 10:**
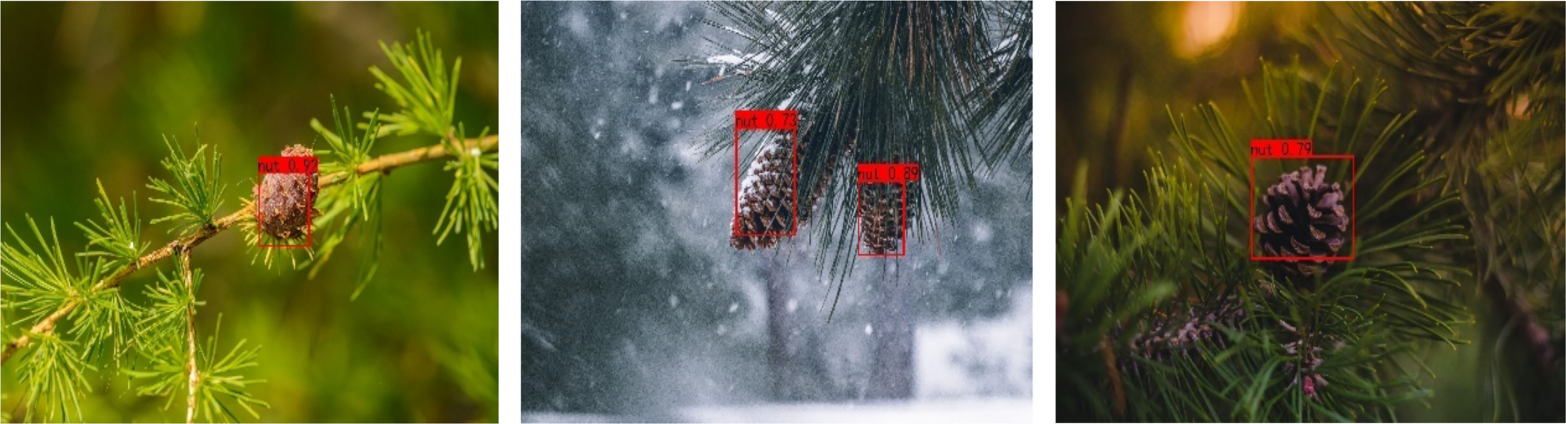
Pine cone detection based on EBE-YOLOV4.

**Figure 11:**
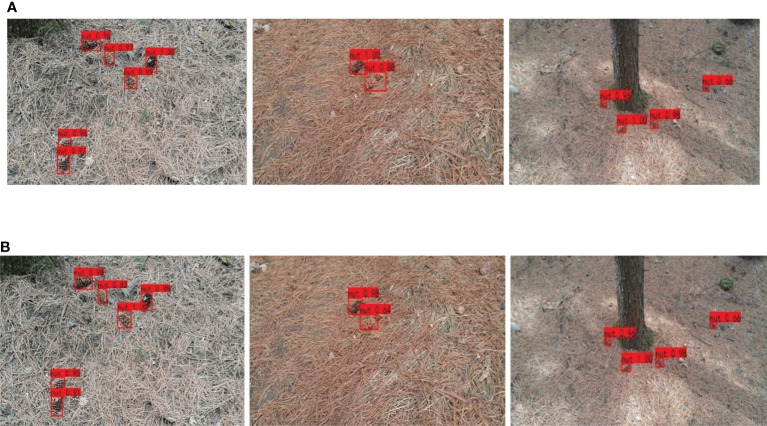
Detection results of YOLOv4 and EBE-YOLOv4:**(A)** Detection based on YOLOv4; **(B)** Detection based on EBE-YOLOv4.

**Figures 10**, **11** are schematic diagrams of the detection of pine cones in forest. **Figure 10** shows the pine cones on the tree, and **Figure 11** shows the pine cones on the ground. It is seen from **Figure 11** that the detection result of EBE-YOLOv4 network is slightly inferior to that of YOLOv4, but it does not affect the final result of recognition.

#### 3.3.2 Ablation experiment

In order to verify the effectiveness and superiority of the model improvement, step-by-step ablation experiments are set up, and the experimental process settings are shown in **Table 3**. Group 0 is the original YOLOv4 network, group 1 adds the EfficientNet-b0 backbone network on the basis of group 0, group 2 adds the BiFPN feature fusion structure on the basis of group 1, group 3 adds ECA-Net module on the basis of group 2, and group 4 introduces H-Swish function on the basis of group 3.

**Table 3:** Ablation experiment design.

Group	YOLOv4	EfficientNet-b0	BiFPN feature fusion	ECA-Net	H-Swish
0	√				
1	√	√			
2	√	√	√		
3	√	√	√	√	
4	√	√	√	√	√

The symbol "√" indicates that the corresponding improvement strategy is adopted in the model, and the absence of the symbol "√" indicates that the corresponding improvement strategy is not adopted.

In addition, based on the optimization of the EfficientNet-b0 backbone network, the neck feature fusion network is compared. The original PANet and the Bi-FPN structure designed in **Table 2** are used, respectively, and the experimental results are shown in **Table 4**. It is seen from **Table 4** that the FPS of EfficientNet-b0-YOLOv4 using the PANet structure is 51.50. After introducing the BiFPN structure of different channels, the FPS of these detection models is significantly improved, and P, R and Mcc do not drop obviously. Among them, the design of 128×3 channels is used in group 3, and the FPS is 65.48, which is slightly lower than that of 66.25 in group 4, but P and R and the comprehensive detection accuracy index Mcc are better than group 4. Therefore, channel 3 is selected as BiFPN feature fusion structure.

**Table 4:** Effectiveness test of neck feature fusion.

Channel design	P	R	AP	F1	Mcc	IT (ms)	FPS (F/S)
PANet	95.03%	84.16%	90.75%	0.892	89.61%	19.41	51.50
1	94.13%	80.06%	89.24%	0.864	87.43%	15.98	62.58
2	93.13%	83.28%	91.29%	0.882	88.79%	16.43	60.86
3	94.11%	79.08%	88.83%	0.861	86.95%	15.17	65.48
4	93.70%	78.59%	87.59%	0.858	85.73%	15.07	66.25

The results of ablation experiment are shown in **Table 5**, in which, Groups 0-2 are the lightweight improvement process, during which, the FPS increased from 37.65 to 65.91, an increase of 75.1%, while P and R decreased from 95.97% and 83.95% to 94.11% and 79.08% respectively, down by 1.9% and 4.9% respectively, and Mcc also decreased from 89.63% to 86.95%, a decrease of 2.7%. Groups 2-4 are the detection accuracy of the optimization model, through which the inference time(IT) slightly increases from 15.17 to 15.60, while the accuracy is significantly improved from 94.11 and 79.08 to 96.25 and 82.72 respectively, and Mcc is improved from 86.95% to 89.23%.

**Table 5:** Statistics of ablation experiment.

Group	P	R	AP	F1	Mcc	IT (ms)	FPS (F/S)
0	95.97%	83.95%	90.70%	0.895	89.63%	26.55	37.65
1	95.03%	84.16%	90.75%	0.892	89.71%	19.41	51.50
2	94.11%	79.08%	88.83%	0.859	86.95%	15.17	65.91
3	94.41%	81.09%	89.26%	0.872	87.90%	15.54	64.34
4	96.25%	82.72%	90.43%	0.890	89.23%	15.60	64.09

Among them, the ECA-Net focuses on improving the index R, and the H-Swish function has a significant improvement effect on both P and R, and the improvement of Mcc is also more obvious.

#### 3.3.3 Lightweight network comparison experiment

To verify the effectiveness and superiority of the method we proposed in this paper, the detection results of YOLOv3, YOLOv4, YOLOv4-tiny and MN3-YOLOv4-Lite (Fu et al., 2021) are compared, and the detection results are shown in **Table 6**. Compared with YOLOv3 and YOLOv4, not only the detection accuracy, but also the FPS is greatly improved, and the improvement ranges from 42.95, 37.67 to 64.09 respectively. As an increase of 49% and 70% is seen, the real-time performance has been significantly improved.

**Table 6:** Comparative experiments.

Network Model	P	R	AP	F1	Mcc	IT (ms)	FPS (F/S)
YOLOv3	94.16%	83.40%	89.42%	0.885	88.21%	23.28	42.95
YOLOv4	95.97%	83.95%	90.70%	0.895	89.75%	26.55	37.67
YOLOv4-tiny	83.68%	74.14%	78.23%	0.784	77.42%	13.58	73.65
MN3-YOLOv4-Lite	95.09%	85.33%	91.02%	0.898	90.11%	17.67	56.59
EBE-YOLOv4	96.25%	82.72%	90.43%	0.890	89.23%	15.60	64.09

Compared with YOLOv4-tiny and MN3-YOLOv4-Lite, the test results are different due to different improvement strategies. YOLOv4-tiny uses the operation of compressing the network depth and reducing the number of output branches, which greatly reduces the amount of calculation, but the feature extraction ability is weakened, and the detection accuracy is reduced. Therefore, the running speed of YOLOv4-tiny is about 9.5 (f/s) higher than the model we proposed in this paper, while the P and R indexes are reduced by 12.6% and 8.6% respectively, the Mcc index is reduced by 11.8%, and the accuracy is significantly reduced. MN3-YOLOv4-Lite introduces Mobilenetv3 and depth convolution. For the model structure and the feature extraction ability are not changed, the accuracy is maintained, but the speed increases low, lower than 7.5 (f/s) of the model we proposed.

## 4 Conclusion

This paper studies the rapid and accurate identification of pine cones in forest. Based on YOLOv4 framework, a lightweight design for rapid detection and a structural optimization strategy for improving accuracy are proposed. By introducing the lightweight backbone network EfficientNet-b0, the neck network adopts the BiFPN feature fusion structure of channel transformation, which reduces the computational complexity of the model. After the feature fusion, a lightweight attention module ECA-Net is added, which improves the model detection accuracy under the premise of adding a small amount of computation. At the same time, the Hard-Swish activation function is used to improve the comprehensive performance of the model. Through the detection experiment of pine cones in forest, the designed lightweight YOLOv4 model has significantly improved performance compared with YOLOv4, YOLOv4-tiny and MN3-YOLOv4-Lite in terms of precision, recall rate and detection speed, and thus realizing faster and more accurate identification of pine cones in forest.

## Data availability statement

The original contributions presented in the study are included in the article/Supplementary Material. Further inquiries can be directed to the corresponding author.

## Author contributions

YZ designed the paper and method and approved the final paper. ZZ wrote the drafting papers and collected the data. DJ and HY made an important revision to the paper. All authors contributed to the article and approved the submitted version.

## Conflict of interest

The authors declare that the research was conducted in the absence of any commercial or financial relationships that could be construed as a potential conflict of interest.

## Publisher’s note

All claims expressed in this article are solely those of the authors and do not necessarily represent those of their affiliated organizations, or those of the publisher, the editors and the reviewers. Any product that may be evaluated in this article, or claim that may be made by its manufacturer, is not guaranteed or endorsed by the publisher.
